# Trifluridine/tipiracil in combination with local therapy may be a favorable option for refractory metastatic colorectal cancer patients: A case report

**DOI:** 10.1097/MD.0000000000022780

**Published:** 2020-10-23

**Authors:** Yu-Lin Lin, Kao-Lang Liu, Been-Ren Lin

**Affiliations:** a Division of Hematology and Oncology, Department of Internal Medicine, Cardinal Tien Hospital, New Taipei City; b Department of Medical Imaging, National Taiwan University Cancer Center, National Taiwan University Hospital and National Taiwan University College of Medicine; c Department of Surgery, National Taiwan University Hospital and National Taiwan University College of Medicine, Taipei, Taiwan.

**Keywords:** Lonsurf, metastatic colorectal cancer, TAS-102, trifluridine/tipiracil

## Abstract

**Rationale:**

Currently, the 5-year survival rate remains poor for patients with metastatic colorectal cancer (mCRC), and the purpose of therapy is to prolong survival while maintaining the quality of life. Trifluridine/tipiracil, an oral drug combining trifluorothymidine and a thymidine phosphorylase inhibitor, is indicated as salvage therapy for mCRC patients who have progressed after all available regimens. Combination of local treatments with systemic therapy such as trifluridine/tipiracil represents an apt management strategy for mCRC patients.

**Patient concerns:**

A 72-year-old man diagnosed with stage IV rectal adenocarcinoma (KRAS mutation) with peritoneal carcinomatosis and liver metastases developed resistance to 2 lines of treatment (bevacizumab/irinotecan/S-1 and bevacizumab/oxaliplatin/HDFL [high-dose 24-hour infusion of 5-fluorouracil and leucovorin regimen]) within 5 months.

**Diagnosis:**

Refractory stage IV rectal adenocarcinoma.

**Interventions:**

Systemic treatment of trifluridine/tipiracil has been given for approximately 15 months in addition to radiotherapy, Yttrium-90 radioembolization, and trans-arterial chemoembolization for peritoneal and liver metastases.

**Outcomes:**

After 15 months, the patient was still taking trifluridine/tipiracil for disease control with a good quality of life.

**Lessons:**

Trifluridine/tipiracil plus other appropriate local therapy may significantly prolong patients survival with a satisfactory quality of life for patients with refractory mCRC. The favorable safety profile of trifluridine/tipiracil renders it a suitable option to be combined with other local therapies for metastatic lesions.

## 1 Introduction

Colorectal cancer (CRC) remains the third most common cancer and is one of the leading causes of cancer-related deaths worldwide.^[1–3]^ The 5-year relative survival rate is about 14% for patients diagnosed with stage IV CRC, in contrast to 90% and 71% for stage I and stage II-III CRC, respectively.^[4]^ For patients with stage IV CRC who develop resistance or are intolerant to first- and second-line therapies, only limited treatment options are currently available.

Both regorafenib (Stivarga; Bayer) and trifluridine/tipiracil (Lonsurf or TAS-102; TTY Biopharm Company Limited, Taiwan/Taiho Pharmaceutical, Japan) were approved by the United States Food and Drug Administration (FDA) and Taiwan Food and Drug Administration (TFDA) to treat patients with metastatic CRC (mCRC) who have been previously treated with fluoropyrimidine-, oxaliplatin- and irinotecan-based chemotherapy, as well as a targeted therapy with anti-vascular endothelial growth factor (VEGF) monoclonal antibodies (bevacizumab/ramucirumab), and if RAS wild-type, anti-epidermal growth factor receptor (EGFR) monoclonal antibodies (cetuximab/panitumumab).^[5,6]^ Although regorafenib and trifluridine/tipiracil are placed on the same position for the treatment algorithm, these 2 drugs differ in their mechanisms of action. While regorafenib is a multi-targeted tyrosine kinase inhibitor that blocks multiple kinases involved in tumor angiogenesis and oncogenesis,^[7]^ trifluridine/tipiracil is an oral combination of trifluridine, a nucleoside metabolic inhibitor, and tipiracil, a thymidine phosphorylase inhibitor, in a molar ratio of 1:0.5. Trifluridine can incorporate into DNA and interferes with DNA synthesis, resulting in inhibition of cell proliferation. In the presence of tipiracil, the anti-tumor activity of trifluridine is augmented through inhibiting its metabolism by thymidine phosphorylase.^[8,9]^

The efficacy of regorafenib and trifluridine/tipiracil have been demonstrated in 4 randomized phase III trials.^[7,10–12]^ Although studies have shown that regorafenib and trifluridine/tipiracil have comparable efficacy, regorafenib was associated with more quality-of-life-impairing toxicities as compared with trifluridine/tipiracil.^[13,14]^ For this reason, the favorable safety profile of trifluridine/tipiracil renders it a relatively more suitable drug to be combined with other treatments in a third-line setting.^[15]^

Herein, we reported a case of metastatic rectal adenocarcinoma in whom the resistance to the first- and second-line treatments developed quickly within 5 months, which implied that the disease was aggressive and rapidly progressive. It is encouraging that he has survived for almost 2 years with a good quality of life after diagnosed with stage IV mCRC and received the initial 2 lines of standard treatment with bevacizumab/irinotecan/S-1 and bevacizumab/oxaliplaitn/infusional 5FU/leucovorin. With this brief case report, we discussed the effectiveness of trifluridine/tipiracil in disease control and its strength in combination with local therapy for refractory mCRC.

## 2 Case presentation

A 72-year-old male with Eastern Cooperative Oncology Group (ECOG) performance status of 1 was originally diagnosed with stage IIIB (cT3N2M0) rectal adenocarcinoma in 2012. Following treatments of concurrent chemoradiotherapy (CCRT) and abdominoperineal resection (APR), the postoperative pathologic finding revealed a stage of ypT2N0M0, and the cancer possessed a KRAS G13D mutation and a wild-type BRAF. There was no evidence of disease recurrence during the subsequent follow-up period.

**Figure 1:**
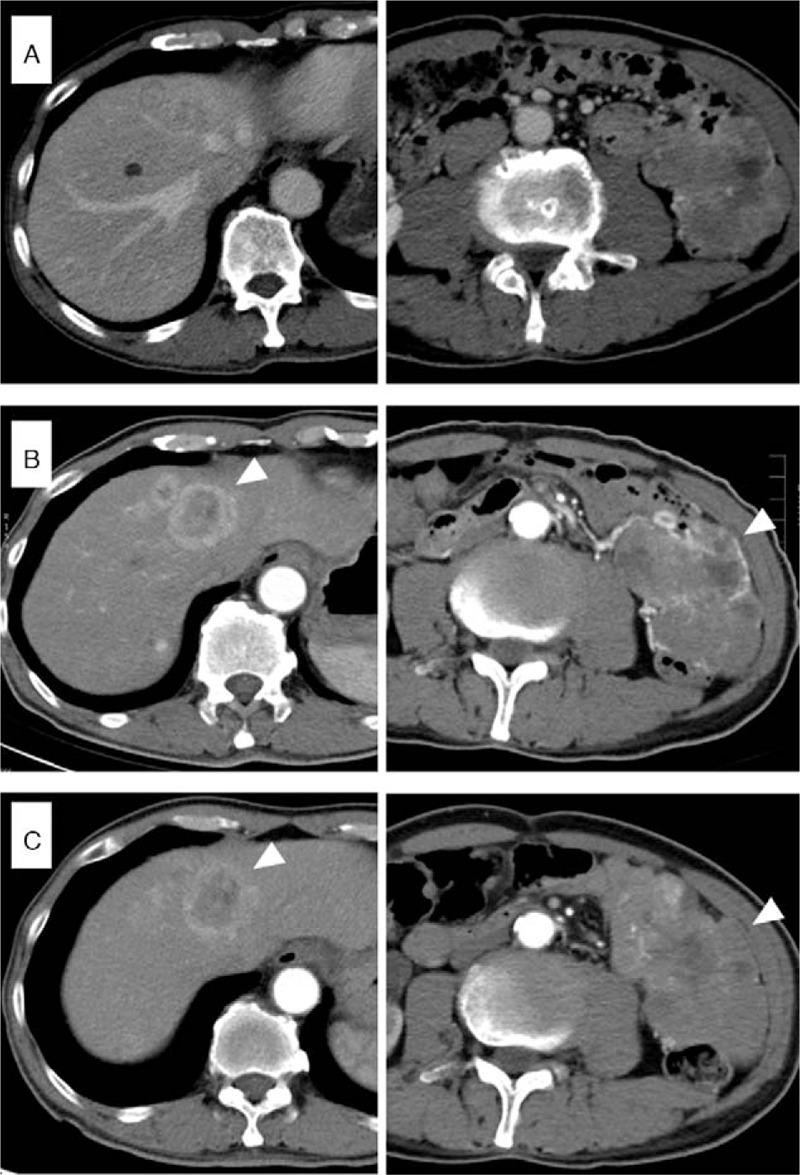
Abdominal computed tomographic images showing liver and peritoneal dissemination. The images show the tumors at the beginning of the treatment (A), following first-line treatment combining bevacizumab, Irinotecan, and S-1 (B), and after second-line treatment of bevacizumab, oxaliplatin, and HDFL (high-dose 24-hour infusion of 5-fluorouracil and leucovorin regimen) (C). Arrowheads indicate tumors with an increase in size.

Nevertheless, in December 2016, the disease had advanced to stage IV with liver metastases and peritoneal carcinomatosis, as confirmed by computed tomography (CT) images (Fig. 1A). Due to disease progression, the patient was enrolled in a phase IV clinical trial and was administered with a combination treatment of bevacizumab, irinotecan, and S-1 (tegafur/gimeracil/oteracil). Unfortunately, disease progression (assessed according to Response Evaluation Criteria in Solid Tumor version 1.1) was observed after 1 month of treatment on day 1 of treatment cycle 3, with an increased size of peritoneal seeding at left abdomen as detected with CT scan (Fig. 1B), and the systemic chemotherapy was then shifted to the second-line regimen combining bevacizumab, oxaliplatin, and HDFL (high-dose 24-hour infusion of 5-fluorouracil and leucovorin regimen). The patient suffered an around 10% loss of body weight and experienced gastrointestinal (GI) bleeding events during the two-lines of treatment, but no active bleeder was identified by enteroscopy. In April 2017, only 3 months after starting second-line treatment, the CT scan indicated an enlarged peritoneal metastasis (Fig. 1C).

**Figure 2:**
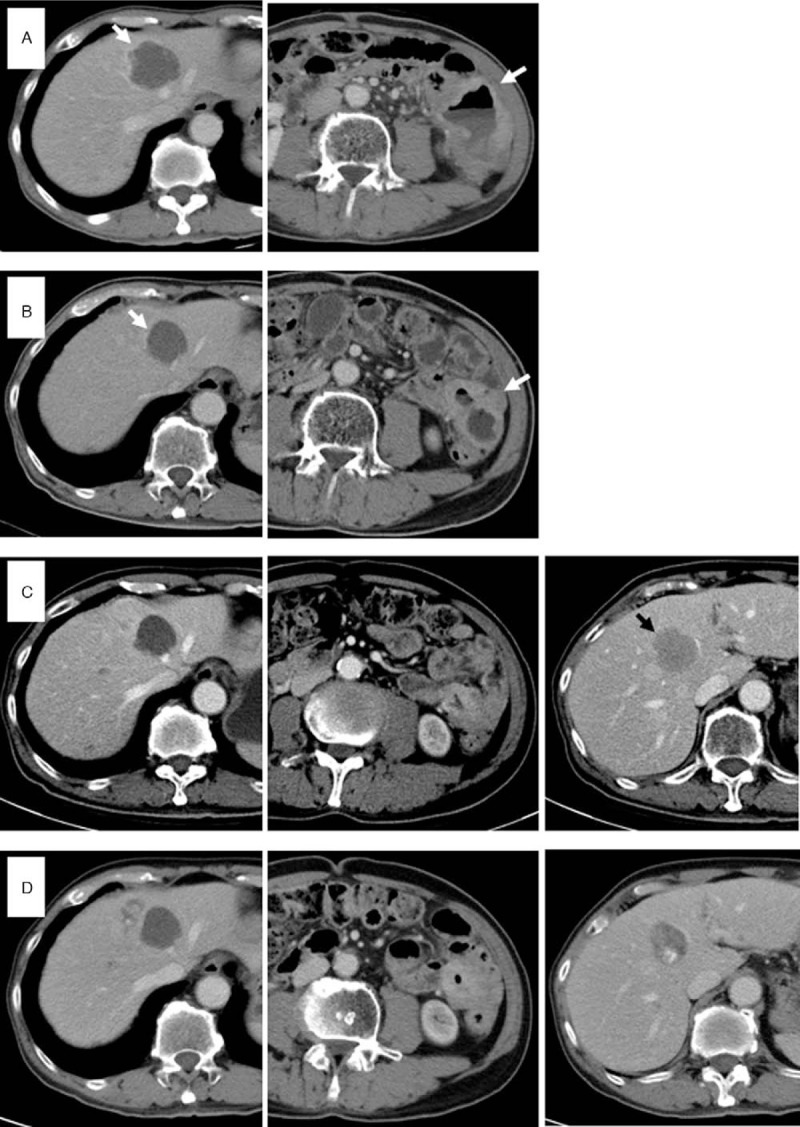
Abdominal computed tomographic images following local treatments for metastatic lesions and systemic trifluridine/tipiracil administration. The images display the tumors following local therapies of radiotherapy and Yttrium-90 radioembolization (A), as well as 4 **(B)**, 7 (C), and 10 (D) months after initiating trifluridine/tipiracil administration. Arrows indicate tumors with a decrease in size and black arrow indicates a new lesion.

Because resection of the left-side peritoneal lesions was not recommended based on the patients condition, the patient underwent local radiotherapy (3600cGy/12Fx) to the left peritoneal carcinomatosis in May 2017, and the symptom of tarry stool was resolved afterward. Subsequently, Yttrium-90 (Y90) radioembolization (2.1GBq) treatment for liver metastases was conducted in early August 2017 due to limited options left for subsequent treatments. After the procedure, most liver metastases in both lobes improved (Fig. 2A). However, 1 viable tumor (5 cm) at segment 5 of the liver was located in the cold area of Bremstrahlung scan, suggesting the need of further trans-arterial chemoembolization (TACE) for this tumor, which was performed 2 months later.

In Taiwan, the National Health Insurance (NHI) only reimburses the treatment of regorafenib for patients with mCRC after they fail standard intravenous chemotherapy with certain targeted therapies (bevacizumab or cetuxitmab if RAS wild-type). However, with concerns of slightly increased bleeding risk that might be caused by regorafenib when the patient had GI bleeding history, trifluridine/tipiracil was given instead of regorafenib at a reduced dosage of 40 mg BID in September 2017. Four months after taking trifluridine/tipiracil, follow-up CT scan showed liver metastases became smaller, which reflected the therapeutic efficacy of the treatments (Fig. 2B). Although the patient experienced grade 2 nausea and anorexia (according to Common Terminology Criteria for Adverse Events version 4.03) after taking trifluridine/tipiracil, there was no significant weight loss at the time.

**Figure 3:**
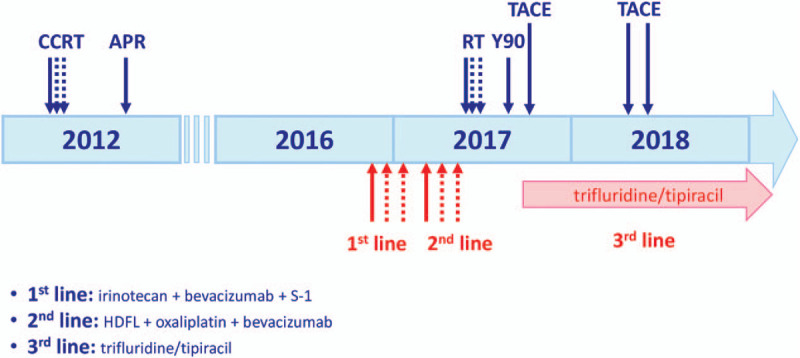
Treatment timeline of the patient. The timeline illustrates the chronology of treatments and responses for the patient. CCRT, concurrent chemoradiotherapy; APR, abdominoperineal resection; RT, radiotherapy; Y90, Yttrium-90; TACE, trans-arterial chemoembolization; HDFL, high-dose 24-hour infusion of 5-fluorouracil and leucovorin regimen.

In late April 2018, a new lesion was observed (Fig. 2C). Due to the progression of the disease and the patient preferred continuing trifluridine/tipiracil rather than switching to regorafenib, the dosage of trifluridine/tipiracil was elevated to 55 mg BID (the recommended dosage for this patient), and another TACE was performed in May 2018. In response to grade 3 neutropenia, trifluridine/tipiracil was later reduced to 55/40 mg BID for the following cycles, which improved the condition of neutropenia to grade 1. During the latest cycle of trifluridine/tipiracil treatment (55 mg BID), the patient had grade 2 neutropenia, and the symptoms of nausea and anorexia were effectively relieved by granisetron. His body weight remained steady with a good quality of life. Although shifting to another regimen has been suggested again, the patient refused and still preferred continuing trifluridine/tipiracil treatment for its manageable adverse reactions. At the last visit, the disease was considered stable based on CT scan (Fig. 2D), and the patient continued trifluridine/tipiracil treatment. The clinical treatments for the patient are summarized as a timeline as illustrated in Figure 3.

## 3 Discussion

Although the age-standardized mortality rate of CRC has been declining in the past 2 decades, the 5-year survival rate in patients diagnosed with advanced CRC remains poor.^[3]^ For mCRC patients who have received standard first- and second-line combination chemotherapy with monoclonal antibodies, the median overall survival in the group of patients receiving subsequent best supportive care (BSC) were only several months.^[16]^ With the exception of some patients with confined and completely resectable tumors, the purpose of therapy for stage IV CRC is generally to prolong life expectancy while maintaining a satisfactory quality of life.

In this report, we described a patient who quickly developed resistance to 2 lines of major treatments. According to the results of recent randomized clinical trials, a median progression-free survival of approximately ten months in patients treated with bevacizumab-combined first-line chemotherapy was reported.^[17,18]^ Even in the subgroup analysis, mCRC patients received FOLFIRI plus bevacizumab as first-line regimen showed a median progression-free survival of 8.9 months (95% CI 7.3–11.4) or longer (9.7 months; 95% CI 8.5–11.2).^[19,20]^ Thus, failing 2 frontline therapies within 5 months suggests the patient had a rapidly progressive disease. Nevertheless, the combined treatment of trifluridine/tipiracil and local therapies showed encouraging therapeutic effects. Until submission of the present report, the patient has survived for over 2 years after the diagnosis of mCRC. In contrast to the median progression-free survival of 2.0 months (95% CI, 1.9 to 2.1) in the trifluridine/tipiracil group reported in the pivotal study,^[10]^ the present case had a stable disease for 7 months after the initiation of trifluridine/tipiracil administration, which may be attributable to the local therapies performed for the metastases during the trifluridine/tipiracil treatment period.

Optimizing the cost-effectiveness is an indispensable part of disease management. The benefits of each treatment option have to be weighed against all possible risks. Following the local radiotherapy for peritoneal carcinomatosis, procedures such as TACE, radiofrequency ablation (RFA), and Y-90 radioembolization are among the choices for treating liver metastases. To maximize cost-benefit ratio based on the patients insurance coverage and to minimize discomfort/suffering of the procedures involved, the patient agreed to receive Y-90 radioembolization under the recommendation of our multidisciplinary team.

For the patient population who have progressed after all available chemotherapeutic regimens, only regorafenib and trifluridine/tipiracil are left as treatment options currently. Based on the results of phase III studies (i.e., the CORRECT study and the RECOURSE study), the median overall survival in the treatment and placebo groups were 6.4 vs 5.0 months (hazard ratio [HR] 0.77; 95% confidence interval [CI] 0.64–0.94; *P* = .0052), respectively, for regorafenib and 7.1 vs 5.3 months (HR 0.68; 95% CI 0.58–0.81; *P* < .001), respectively, for trifluridine/tipiracil.^[7,10]^ Although the 2 drugs provide survival benefits of a similar magnitude, the adverse reaction profiles are in favor of trifluridine/tipiracil.^[13,14,21]^ After a thorough explanation of possible and anticipated risks, the patient chose to receive trifluridine/tipiracil as his third-line treatment. During the 15-month treatment of trifluridine/tipiracil, the dosages of trifluridine/tipiracil were modified according to the patients physiological condition and were greatly affected by the patients private insurance policy. As mentioned previously, the Taiwan NHI only reimburses regorafenib, but not trifluridine/tipiracil, in certain conditions. The average cost of trifluridine/tipiracil is approximately $6,450 per month. In this regard, despite that the recommended dose for this patient was approximately 55 mg BID based on his body surface area, the starting dosage was only 40 mg BID due to aforementioned reasons. Without considering the frequency, the patient received an overall of 80.52% of the recommended dose. When the treatment duration and frequency of administration were considered, he only received 51.24% of the recommended dose. It suggests that, for patients who possess polychemotherapy refractory mCRC, and in the presence of KRAS mutation, an appropriate combination of local and systemic treatments may provide survival benefit with a good quality of life, despite the dosage of trifluridine/tipiracil was considerably less than its recommended dose. At the last visit, the patient continued trifluridine/tipiracil treatment at 55 mg BID, and the disease was considered stable.

The patients body weight declined substantially during the first 2 lines of chemotherapy, after which the body weight increased and maintained stable during the trifluridine/tipiracil treatment period, despite that the patient experienced trifluridine/tipiracil-related adverse events (AEs) including nausea, decreased appetite, and neutropenia (these AEs were among the most common reported adverse reactions to this medication).^[10]^ In the RECOURSE study, neutropenia, leukopenia, anemia, and thrombocytopenia of grade 3 or higher occurred in 38%, 21%, 18%, and 5% of the patients receiving trifluridine/tipiracil, respectively.^[10]^ The present case, however, had only experienced 1 event of grade 3 neutropenia after the dosage of trifluridine/tipiracil was increased to 55 mg BID for the first time. Interestingly, a study conducted by Kasi et al revealed that patients who developed neutropenia of grade 2 or worse at 1 month after starting trifluridine/tipiracil had a significantly longer progression-free survival and overall survival, indicating that the myelosuppression effect of neutropenia could act as a predictive factor.^[22]^ Giuliani and Bonetti recently showed that the onset of severe neutropenia (grade 3 or worse) at the first-cycle was significantly associated with longer median overall survival.^[23]^ Moreover, Hamauchi et al also suggested that trifluridine/tipiracil-induced neutropenia was associated with better disease control rate, and could be a surrogate marker for adjusting adequate trifluridine/tipiracil dose.^[24]^

## 4 Conclusion

The National Comprehensive Cancer Network (NCCN) guideline version 3.2018 recommends trifluridine/tipiracil, regorafenib, or best supportive care for mCRC patients refractory to first- and second-line treatments. While both trifluridine/tipiracil and regorafenib are considered salvage regimens, the side effect profile of trifluridine/tipiracil renders it a more favorable option to be applied in combination with other local therapies in the third-line setting.^[13,14]^ Moreover, the patients prescribing trifluridine/tipiracil were more likely to adhere to their treatment as compared with those taking regorafenib.^[25]^ Collectively, the case reported here suggests that trifluridine/tipiracil plus local treatments might be a feasible therapeutic combination for patients who have progressed after 2 lines of major therapy.

## 5 Consent

The case report has been waived for the ethical approval by the standard of the Research Ethics Committee of National Taiwan University Hospital. Written informed consent was obtained from the patient before the publication of this case report and accompanying images.

## Author contributions

**Conceptualization:** Yu-Lin Lin.

**Formal analysis:** Kao-Lang Liu, Been-Ren Lin.

**Resources:** Kao-Lang Liu, Been-Ren Lin.

**Supervision:** Been-Ren Lin.

**Visualization:** Kao-Lang Liu, Been-Ren Lin.

**Writing – original draft:** Yu-Lin Lin.

**Writing – review & editing:** Yu-Lin Lin, Kao-Lang Liu, Been-Ren Lin.
